# Zinc Finger Readers of Methylated DNA

**DOI:** 10.3390/molecules23102555

**Published:** 2018-10-07

**Authors:** Nicholas O. Hudson, Bethany A. Buck-Koehntop

**Affiliations:** Department of Chemistry, University of Utah, Salt Lake City, UT 84112-0850, USA; u1082423@utah.edu

**Keywords:** zinc finger, methyl-CpG binding proteins, DNA methylation, epigenetics, protein-DNA interactions

## Abstract

DNA methylation is a prevalent epigenetic modification involved in regulating a number of essential cellular processes, including genomic accessibility and transcriptional outcomes. As such, aberrant alterations in global DNA methylation patterns have been associated with a growing number of disease conditions. Nevertheless, the full mechanisms by which DNA methylation information is interpreted and translated into genomic responses is not yet fully understood. Methyl-CpG binding proteins (MBPs) function as important mediators of this essential process by selectively reading DNA methylation signals and translating this information into down-stream cellular outcomes. The Cys_2_His_2_ zinc finger scaffold is one of the most abundant DNA binding motifs found within human transcription factors, yet only a few zinc finger containing proteins capable of conferring selectivity for mCpG over CpG sites have been characterized. This review summarizes our current structural understanding for the mechanisms by which the zinc finger MBPs evaluated to date read this essential epigenetic mark. Further, some of the biological implications for mCpG readout elicited by this family of MBPs are discussed.

## 1. Introduction

The most prevalent epigenetic modification found on DNA is the addition of a methyl group at the 5-position of cytosine bases. In mammals, the addition of this reversible chemical modification most commonly occurs in the context of CpG dinucleotides and is referred to as DNA methylation. Genomic DNA methylation patterns are primarily established during development by the de novo DNA methyltransferases (DNMT), 3A and 3B, and heritably maintained during DNA replication by DNMT1 [1,2,3,4]. Across the genome, most CpG sites are methylated (mCpG), with the exception of GC-rich stretches termed CpG islands (CGIs), found in ~70% of all gene promoters [5]. While DNA methylation inherently represents a seemingly small alteration in the chemical signature, this epigenetic alteration plays a significant role in regulating a number of key processes in both normal cell function as well as disease conditions. In the early stages of development, DNA methylation is essential for regulating genomic imprinting, X-chromosome inactivation, and cellular differentiation during embryogenesis [6,7,8]. Misregulation of DNA methylation patterns has thus been linked to several neuro-developmental diseases, including Rett, fragile X, and ICF (immunodeficiency, centromeric instability and facial anomalies) syndromes [9,10].

Beyond its role in development, DNA methylation functions to maintain overall genomic stability, preserve cellular identity, and control gene expression by working in conjunction with specific patterns of post-translational modifications (PTMs) on histone proteins to regulate chromatin accessibility and nucleosomal positioning [6,9,11,12]. Aberrant alterations in genomic DNA methylation patterns have consequently been associated with a multitude of disease conditions. In particular, it has become evident that nearly all cancers harbor significant alterations in their DNA methylation patterns that correlate with disease phenotype [13,14,15,16]. Specifically, in many cancers, a global decrease in genomic CpG methylation concomitant with hyper-methylation of promoter CGIs is associated with abnormal proliferation of cells preceding tumorigenesis [9,14,17,18]. Further, DNA methylation patterns have been observed to vary with tumor type and grade [19], promote tumor heterogeneity [20], and be clonally inherited [21]. In addition to cancer, there is increasing evidence that misappropriated DNA methylation patterns are associated with a variety of other disease conditions [22,23,24], including: Metabolic disorders (e.g., obesity and type II diabetes) [25,26,27,28]; autoimmune diseases (e.g., type I diabetes, lupus, and rheumatoid arthritis) [29,30,31]; and neurological diseases (e.g., Parkinson’s, Huntington’s, and Alzheimer’s) [32]; as well as psychiatric disorders (e.g., depression, anxiety, and schizophrenia) [33,34]. Further, evidence of age related causative DNA methylation alterations in disease promotion have been proposed [35]. While there is significant evidence implicating a role for DNA methylation in a multitude of disease conditions, the full mechanisms by which DNA methylation information is interpreted and translated into genomic responses is not yet fully understood.

In the context of promoter CGIs, DNA methylation is typically associated with inducing transcriptional repression at the target site. Two primary mechanisms by which DNA methylation is believed to elicit gene silencing is by either preventing or promoting transcription factor (TF) binding [11,36]. Specifically, the addition of a methyl group on cytosine bases can affect TF readout at CpG sites by introducing local perturbations to the DNA structure as well as presenting a hydrophobic edge within the major groove binding interface [37,38,39]. These combined alterations to the DNA can have an overall repulsive effect on the ability of TFs to recognize their cognate DNA binding sequences, leading to transcriptional inhibition [40,41,42,43,44]. Alternatively, these modifications can facilitate the recruitment of TFs that have preferential selectivity for mCpG sites, termed methyl-CpG binding proteins (MBPs); a subject that has been extensively reviewed [45,46,47,48,49,50,51,52,53]. There is evidence to suggest that direct mCpG occupation by some MBPs can recruit chromatin remodeling enzyme complexes to the target site that subsequently leads to alterations in chromatin architecture and transcriptional outcomes [54,55,56,57,58,59,60]. In this capacity, MBPs function as essential functional intermediaries in the gene regulation process. As such, there has been significant interest in mechanistically discerning how these MBPs select and interpret DNA methylation signals, as well as evaluating their potential as novel therapeutic targets. Indeed, it has been demonstrated that the depletion of MBPs in the cancerous context can re-activate transcriptional activity at repressed promoters without altering the methylation status [61,62,63,64]. This implies that when mediated by MBPs, DNA methylation may be necessary, but not sufficient, to elicit gene silencing.

In contrast to most hyper-methylated promoter CGIs [65,66], high levels of DNA methylation at gene body CGIs have been correlated with actively transcribed genes [67,68,69], suggesting a more intricate role for DNA methylation in gene regulation. Gene body DNA methylation sites have been proposed to function as alternative promoters during transcription and to regulate differential splicing [70,71,72,73,74,75]. Further, MBPs have been shown to associate with methylated CGIs at both promoters and exons in embryonic stem and cancer cells [76,77]. Interestingly, gene body binding did not impede transcriptional elongation when preceded by an active promoter [76,77]. More recently, it has been proposed that MBP occupation at non-promoter mCpG loci may indirectly guide TFs that are insensitive to CpG methylation to their consensus motifs within non-methylated promoter CGIs [78,79]. Combined, it is evident that MBPs are essential factors in mediating methyl-dependent gene regulation, though mechanistic insight for many of their functions remains to be fully elucidated.

For many years, the identified TF MBPs have been confined to the methyl-CpG binding domain (MBD) and Cys_2_His_2_ zinc finger (ZF) containing families. However, more recently, a series of in vitro high-throughput strategies have been utilized to identify additional TF protein families that appear to harbor mCpG binding capabilities [80,81,82,83]. Structural insight for some of these newly discovered MBP classes, including the basic leucine-zipper (bZIP) [84] and homeodomain [82] proteins, have become available, though further in vitro and in cell characterization/validation for most of the discovered systems is still needed. The MBD family was the first identified class of MBPs discovered [85,86] and, as such, a significant body of research has provided insight for the mechanisms of mCpG recognition, roles in methyl-dependent transcriptional regulation, as well as the overall impact of this protein family on cellular functions. Several reviews have been dedicated to these topics [87,88,89,90,91,92,93,94]. The overall contributions from ZF MBPs in reading this essential epigenetic mark and translating the encoded information into down-stream cellular responses have been comparatively reviewed far less. Hence, our goal here is to provide a current review of the structural evidence that has afforded mechanistic insight for how ZF MBPs distinguish mCpG target sites. Further, we will summarize some of the biological implications for mCpG readout elicited by the currently structurally validated ZF MBPs.

## 2. Mechanistic Recognition of mCpG Sites by Zinc Finger Proteins—Structural Perspective

### 2.1. Structural Insight for ZF Recognition of Methylated DNA

**Figure 1:**
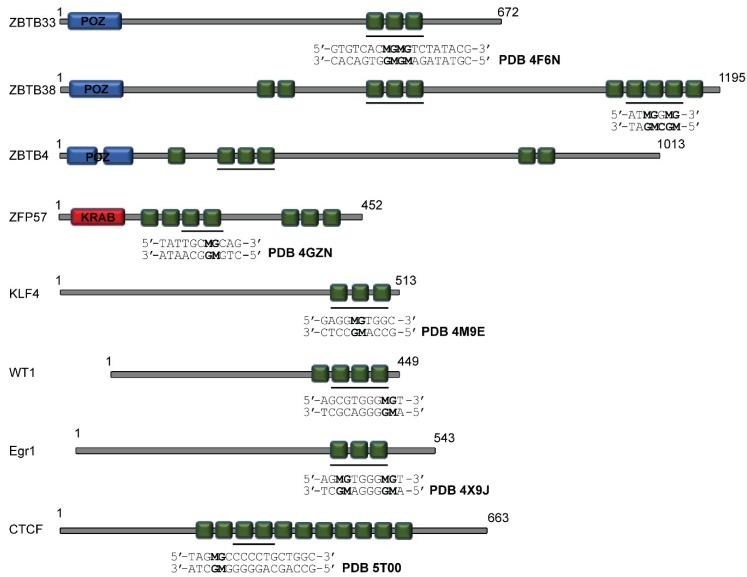
Domain organization for the characterized zinc finger (ZF) methyl-CpG binding proteins (MBPs). For each protein, the ZFs shown to have methyl sensitivity are underlined and the target DNA sequence utilized for high-resolution structural investigations are depicted alongside the Protein Data Bank (PDB) identification for the structures shown in Figure 2. M refers to methylated cytosine. POZ: Pox virus and zing finger domain; KRAB: Krüppel-associated box.

The Cys_2_His_2_ ZF scaffold is one of the most abundant DNA binding motifs found within the human TF proteome. Each modular Cys_2_His_2_ ZF has a core domain comprised of two β-strands that pack against an α-helix (ββα-fold), which is stabilized by tetrahedral coordination of a Zn^2+^ atom through the side chains of two cysteine and two histidine residues [95,96]. In specific DNA recognition, the α-helix orients along the major groove and utilizes resides at specific positions (−1, +2, +3, and +6, relative to the start of the helix) to generally make base contacts within a three base pair region [95,96]. Many proteins use tandem arrays of these ZF motifs to expand sequence-specificity for a given DNA consensus site. Irrespective of the abundance of Cys_2_His_2_ ZF containing proteins, only a few high-resolution structures for TFs containing this structural feature that are capable of conferring selectivity for mCpG over CpG sites have become available in recent years. Currently, these ZF TFs include ZBTB33 (also known as Kaiso) [97,98], Zfp57 [99], Klf4 (Krüppel-like factor 4) [100,101], WT1 (Wilms’ tumor protein 1) [102], Egr1 (growth response protein 1; also known as Zif268) [102,103], and CTCF (CCCTC-binding factor) [104] (Figure 1), the structures of which have begun to provide mechanistic insight for how ZF MBPs can recognize this essential epigenetic mark. 

**Figure 2:**
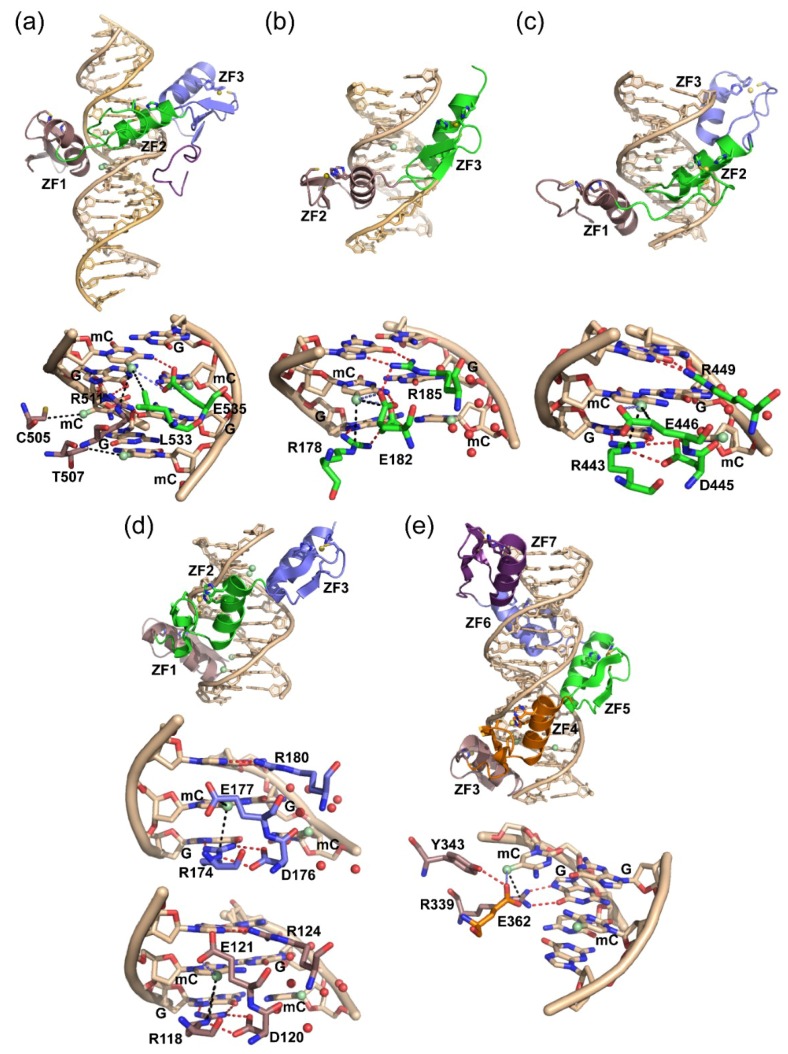
High-resolution crystal structures for members of the ZF MBP family in complex with their respective methylated DNA targets. (**a**) Human ZBTB33 in complex with a methylated DNA sequence derived from the *E*-*cadherin* promoter (PDB 4F6N); (**b**) mouse Zfp57 in complex with a methylated DNA sequence present in imprinting control regions (PDB 4GZN); (**c**) mouse Klf4 (Krüppel-like factor 4) in complex with its cognate methylated DNA sequence (PDB 4M9E); (**d**) human Egr1 (growth response protein 1) in complex with its cognate methylated DNA sequence (PDB 4X91); (**e**) human CTCF (CCCTC-binding factor) in complex with a methylated version of its core recognition sequence (PDB 5T00). Red spheres indicate water molecules. Red dotted lines denote classical hydrogen bond interactions; blue dotted lines indicate CH···O type hydrogen bonds, and black dotted lines designate van der Waals interactions. For each zoomed-in image, the amino acid side chain color designation matches that of the ZF from which it is derived in the full structural image depicted above.

From the currently available structures, it has become evident that a common mode of ZF recognition for mCpG sites involves the usage of key arginine and glutamate residues (Figure 2) [105,106,107,108]. Specifically, in each of these ZF:methylated DNA complexes, an arginine is involved in classical hydrogen bonding interactions with one (Zfp57, Klf4, WT1, Egr1, CTCF) or two (ZBTB33) of the 3′-G residues. This further positions the arginine side chain to make van der Waals contacts with the 5′-mC methyl group on one DNA strand. This designated ‘mC-Arg-G triad’ [107] is also utilized by the MBD family of MBPs, and has been shown to play a significant role in stabilizing the binding interaction as well as affording discrimination capability between mC and C [109]. Intriguingly, while all of the ZF MBPs utilize this mC-Arg-G triad in mCpG recognition and in vitro exhibit low nM binding for mCpG containing sequences (<200 nM), their preferential ability to distinguish mCpG over CpG sites is quite variable. Klf4, WT1, and CTCF each have a ZF domain that has been shown in vitro to exhibit marginal (~1.5–1.8-fold) selectivity for mCpG over CpG [101,102,104]. Depending on the study, Egr1 has been shown to be completely insensitive or have an ~3-fold sensitivity for mCpG over CpG sites [102,103]. In contrast, both ZBTB33 and Zfp57 exhibit significantly higher selectivity for their methylated DNA targets (≥20-fold relative to CpG) [98,99]. This affinity differential between the ZF MBPs appears to be significantly correlated with the extent to which the conserved glutamate residue is involved in recognition of the mC.

ZBTB33 and Zfp57 exhibit the highest selectivity for mCpG sites and in each case, the glutamate residue is involved in classical hydrogen bonding interactions between the glutamate carbonyl oxygens and the N4 atom of one (Zfp57) or both (ZBTB33) of the mCs, as well as interactions between the glutamate side chain and the mC methyl (Figure 2a,b). Of particular note, the glutamate residue is able to make CH···O type hydrogen bonds with the methyl group of one (Zfp57) or both (ZBTB33) of the mCs. Due to the significant number of contacts between the core glutamate and two cross-strand mCs, it is unsurprising that mutation of this residue to an alanine in ZBTB33 abolishes DNA binding [98]. In contrast, mutation of the correlative glutamate in Zfp57 to an alanine showed no difference in DNA binding capability [99]. Closer examination of the Zfp57:methylated DNA structure reveals that the glutamate residue adopts two conformations, one that is more idealized for mC recognition, and one that is better suited for positioning another arginine residue for making hydrogen bonds with a guanine base outside of the mCpG core (Figure 2b). It may be that ZBTB33 is overall better able to spatially coordinate optimal recognition of mCpG sites by utilizing two different α-helices to contribute the arginine and glutamate residues, unlike Zfp57 where both residues are positioned within the same α-helix.

As discussed above, all of the remaining ZF MBPs, including Klf4, WT1, Egr1, and CTCF, have at least one ZF that is either indifferent or has a marginal selectivity for mCpG over CpG sites. For each of these proteins, the glutamate residue is positioned such that it is not capable of forming classical hydrogen bonding interactions with the N4 atoms of the mCs, and is only able to contribute either van der Waals interactions from the side chain and/or CH···O type hydrogen bonding interactions via the carbonyl oxygens with the mC methyl groups (Figure 2c–e). In the cases of Klf4, WT1, and Egr1, there is a conserved aspartate residue preceding the glutamate that simultaneously stabilizes the arginine side chain for recognition of the 3′-G and provides a weaker electrostatic interaction with the mC N4 atom through one of its carbonyl oxygens (Figure 2c,d). Similar to ZBTB33, CTCF also utilizes two different ZF helices to provide the key arginine and glutamate residues for mCpG recognition, however, the glutamate side chain position is fixed such that it is unable to make a classical hydrogen bond with the mC N4 atom (Figure 2e). This seems to be in part due to interactions from a neighboring tyrosine, which positions the glutamate for making a CH···O hydrogen bond with the mC methyl group, but only affords the glutamate an opportunity to form a weaker electrostatic interaction with the mC N4 atom. Combined, these structures suggest that the loss of a base-specific hydrogen bonding interaction between the glutamate and mC plays a significant role in the observed reduced selectivity for mC over C. Furthermore, Klf4 has been shown to bind equally well to targets that contain interchangeable mCpG or TpG base steps [100]. Given that the glutamate interactions are limited to aliphatic side chain contacts with the methyl at the 5-position of either the mC or T base, the inability to discriminate between these bases is unsurprising. In contrast, ZBTB33 is also capable of recognizing both mCpG containing targets as well as a sequence specific motif containing a TpG site (termed the Kaiso binding site (KBS)) [97,110,111]. However, in this case, the glutamate is still within hydrogen bonding distance of the thymine O4, suggesting that the protonation state of the glutamate must be altered to accommodate recognition of the thymine base, and that ZBTB33 specifically selects for both mCpG and TpG containing targets [97].

Another emerging trend observed for ZF MBPs is that the binding at mCpG palindromic sites is asymmetric [106]. For each of the ZF MBPs, except ZBTB33, all the arginine and glutamate interactions predominate at one mCpG (Figure 2b–e). In each of these cases, the remaining palindromic mC is surrounded by an ordered water layer. It has been established that the methyls of mCs are significantly hydrated [112,113], and that this hydration can contribute to the overall binding energetics for MBP recognition of mCpG sites [99,114,115]. In the case of ZBTB33, the glutamate makes cross-strand interactions with both mCs displacing any ordered water molecules around the mC methyl groups. However, the arginine side chain and a proximal leucine residue contribute additional van der Waals contacts for only one mC (Figure 2a), which appears to establish the overall strand preference. Indeed, each of the ZF MBPs has been shown to have similar binding affinities for strand specific hemi-methylated DNA relative to symmetrically methylated DNA sites [97,98,99,101,102]. The physiological consequence of this asymmetric binding capability for each of these proteins is not yet fully understood.

**Figure 3:**
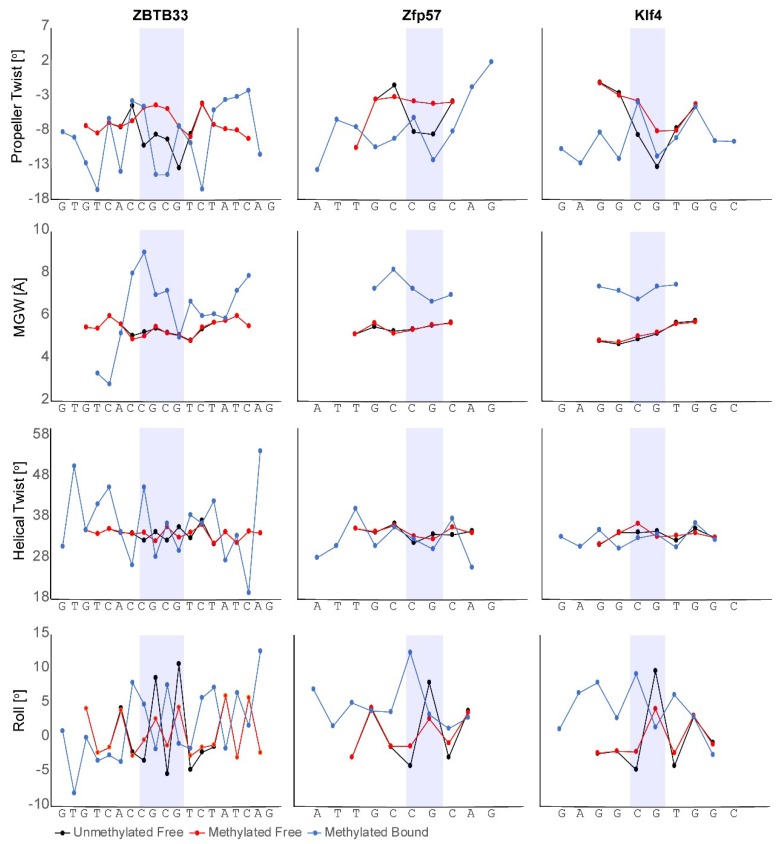
DNA shape analyses for the DNA targets of ZBTB33, Zfp57, and Klf4 used for crystallography in their free unmodified CpG (black), free methyl-CpG (red), and methyl-CpG protein complexed (blue) forms. The blue rectangles are centered on the core CpG dinucleotides.

In addition to base specific readout, the role of local DNA shape is an essential factor in defining the selectivity of a protein for a given DNA target [39,116,117,118]. Recently, it has been demonstrated that methyl addition to cytosine bases within the context of duplex B-form DNA results in significant perturbations to the local DNA shape predominated by changes in roll and propeller twist [38]. Furthermore, these induced local DNA structural changes have been shown to influence protein recruitment and binding to target genomic sites [37,38]. Thus, based on the available structural information, we sought to determine what impact DNA shape alterations may have on mCpG recognition by ZF MBPs. Our analysis was limited to evaluation of the ZBTB33, Zfp57, and Klf4 methylated DNA complexes as the mCpGs were centrally localized within the target DNA sites for these systems. As structures for the DNA targets in their free forms are not available, methyl-DNA shape [38] was first utilized to determine the effect of methylation on each of the target DNAs utilized in the complex structures. As previously observed [38], methylation at each of the CpG sites resulted in minimal changes to the helical twist and minor groove widths (MGWs), but induced larger changes in both roll and propeller twist (Figure 3). In particular, for all of the DNA sequences, methylation induced a site specific decrease and subsequent increase of the roll at the 5′-mC and 3′-G positions, respectively (Figure 3). Further, the degree of propeller twist was decreased upon methylation across the CpG site for all three DNA sequences evaluated.

To determine the impact of protein binding, CURVES+ [119] was utilized to discern how these DNA shape parameters were altered upon complex formation. For each protein:DNA complex, the magnitude of the roll at the 5′-mC and 3′-G positions were substantially increased and marginally reduced, respectively (Figure 3). These combined observations suggest that the specific directional alterations in the degree of roll upon cytosine methylation may be a common mode of DNA shape alteration needed to facilitate ZF MBP binding. No clear pattern for propeller twist alterations were evident across the three protein:DNA complexes, while all three bound DNA targets exhibited a noticeable increase in MGW. For the Zfp57 and Klf4 methylated DNA complex structures, alterations in helical twist were minimal, whereas in the ZBTB33 methylated DNA complex, there was a noticeable increase in helical twist angles for the 5′-mCs and a decrease for the 3′-Gs (Figure 3). This differential impact on helical twist angles upon protein binding may be correlated with the fact that the key arginine and glutamate residues of ZBTB33 make cross-strand base-specific contacts, while these same residues in Zfp57 and Klf4 contact only one half of the mCpG palindrome. Finally, from the CURVES+ analysis, it was determined that the overall deformation in DNA bend from B-form was 21.9°, 11.2°, and 4.2° after ZBTB33, Zfp57, and Klf4 binding, respectively. This observation suggests that the proteins exhibiting a higher methyl selectivity induce a larger global bend in the DNA, likely to facilitate base specific contacts at the core mCpG site relative to the less selective binders. Though more high-resolution structures are required to confirm this.

### 2.2. Additional Methyl-Selective ZF TFs and Alternative Modes of mCpG Recognition

ZBTB33 represents the founding member of the ZBTB family of MBPs, which also includes ZBTB4 and ZBTB38 [120]. All three proteins share a conserved set of three Cys_2_His_2_ ZFs that are responsible for methylated DNA recognition [120] (Figure 1). Importantly, while there are not yet high-resolution structures for ZBTB4 and ZBTB38 in complex with their respective methylated DNA binding targets, all of the core residues involved in ZBTB33 recognition of methylated DNA are conserved. Indeed, similar to ZBTB33, mutation of the key glutamate within ZBTB4 to an alanine abolished binding to methylated DNA [121]. Further, in vitro and in cell evidence suggests that both ZBTB4 and ZBTB38 are also highly selective for mCpG sites over CpG [120,121,122,123,124]. Finally, it has been established that similar to ZBTB33, both ZBTB4 and ZBTB38 exhibit bimodal DNA binding by also recognizing a sequence-specific TpG containing motif [120,121,125].

**Figure 4:**
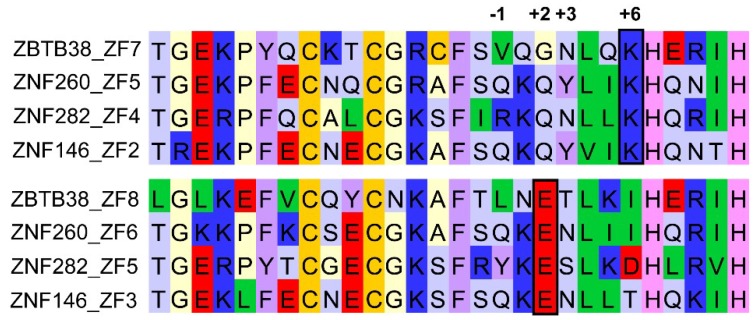
Sequence alignment for additional ZF proteins that share the conserved lysine/glutamate residue pair (boxed) that was shown for the C-terminal ZFs of ZBTB38 to participate in selective mCpG recognition.

In addition to the three conserved ZFs, ZBTB4 and ZBTB38 each have multiple N- and C-terminal ZFs (Figure 1), the functions of which have yet to be fully characterized. Recently it has been demonstrated that a sub-set of the five C-terminal ZFs of ZBTB38 also participate in methyl-selective DNA recognition both in vitro and within the cell [124] (Figure 1). Specifically, it was determined that the middle three ZFs (ZFs 7–9) are necessary and sufficient for mCpG recognition, though one or both of the additional ZFs (ZF6 and/or ZF10) are needed to confer optimal affinity during DNA binding. However, unlike any of the other ZF MBPs characterized to date, it was determined that while ZFs 7 and 8 had the key glutamate residue, they were lacking the core arginine required for mCpG recognition. Rather, a lysine residue in ZF7 was found to be substituted at the +6 position typically occupied by the arginine (Figure 4). Systematic mutation of both the lysine and glutamate residues significantly impacted DNA binding, indicating that these two residues are required for mCpG recognition [124]. This is the first evidence of a lysine residue playing a surrogate role for arginine in ZF recognition of mCpG sites, and suggests that an alternative mode for ZF recognition of mCpG exists.

To determine whether other ZF containing proteins harbor this same lysine/glutamate pair, a Basic Local Alignment Search Tool (BLAST) search using ZFs 7–8 of ZBTB38 was conducted. Three other ZF proteins, including one Krüppel-associated box (KRAB) domain protein (ZNF282), were identified (Figure 4). The biological functions for these three factors are not well characterized, though it would be interesting to evaluate their capacity for methyl-selective DNA recognition. Finally, it is important to note that a number of additional ZF proteins have been identified from recent high-throughput studies to potentially have preferential mCpG binding capacity [80,81,82,83]. Characterization for these additional ZF proteins is needed to determine whether the mechanisms by which ZF proteins can preferentially select for methylated DNA targets needs to be expanded further.

## 3. Physiological Consequence of Methyl Sensitivity

From in vitro biochemical and structural insight, it is evident that the ZF MBPs bind to mCpGs in a sequence-specific context. However, these essential pieces of information are unable to inform on the cellular genomic distributions and functions for these TFs. In recent years, there has been an increasing interest in correlating mechanistic in vitro findings with target selectivity within the cell [118]. In general, determining TF distributions at target sites in the cell is far more complicated due to the fact that typically numerous copies of a consensus motif are present in the genome, but accessibility to these regions can be differential between cellular contexts. Further, binding to a given loci may or may not lead to a cellular response, which is again likely to be cell specific. Nevertheless, the rapidly increasing advances in ChIP-based technologies as well as high-throughput sequencing methodologies have afforded the capability to begin evaluating the cellular readout of methylated DNA targets for many of the structurally characterized ZF MBPs. Here, we briefly correlate the in vitro structural findings for mCpG sensitivity with the potential cellular relevance for each ZF MBP.

### 3.1. ZBTB33, ZBTB4, and ZBTB38

Through their conserved set of three Cys_2_His_2_ set of ZFs, each of the three members of the ZBTB MBP family has been shown to bind to both mCpG as well as sequence specific TpG containing sites [111,120,121,122,123,124,125,126,127]. For ZBTB33, this bimodal DNA binding capacity has provided the capability of functioning to either up- or down-regulate transcription depending on the gene context [128,129,130]. Further, each protein appears to function in an analogous manner to the MBD family of TFs in that direct promoter occupation recruits chromatin remodeling complexes, which subsequently alters transcriptional outcomes [58,59,60,131]. A significant body of work combining in vitro and in cell analyses has implicated the functions of these proteins in transcriptionally regulating genes associated with a number of core cellular functions; particularly in the context of cancer. Of the three family members, ZBTB33 is the best characterized. In particular, ZBTB33 transcriptional activities have been associated with regulating cellular proliferation [128,129,132,133,134], apoptosis [134,135,136], and migration/invasion [137,138,139,140] in various cancer models. Further, ZBTB33 has been shown to be overexpressed in aggressive forms of breast and prostate cancer tumors [137,139,141,142,143]. In several cancers, down-regulation of ZBTB4 correlates with disease advancement [59,144,145] through several mechanisms, including supporting p53-dependent anti-apoptotic activity [59], up-regulating oncogenic proteins [144,145,146], and increasing genomic instability [147]. The evaluation of ZBTB38 in cancer has been limited, though several studies have correlated ZBTB38 transcriptional activities with disease relevant pathways, such as apoptosis [122,148], proliferation [149], and differentiation [123,125,150].

Nevertheless, the analysis of publicly available chromatin immunoprecipitation sequencing (ChIP-seq) data for ZBTB33 determined that it predominantly localizes to a palindromic CpG containing sequence (5′-TCTCGCGAGA-3′) in the non-methylated state [151]. Moreover, no localization for ZBTB33 at any KBS loci was observed, though this sequence motif is highly represented within the human genome, and ZBTB33 binding at this site is not dependent on modification status. These ChIP-seq findings are in contrast to the mode of DNA recognition delineated from structural investigations [97,98,106], as well as the expanding body of literature that demonstrates by ChIP-quantitative PCR (ChIP-qPCR) or -semiquantitative PCR (ChIP-sqPCR) that in cell, ZBTB33 directly localizes to promoters through KBS and/or selective mCpG binding and regulates transcription [61,128,129,130,134,135,136,137,139,143,152]. Notably, most of these ChIP-based studies utilized the same antibody as the ChIP-seq experiments. It has been suggested that the lack of ZBTB33 occupation at mCpG sites from the ChIP-seq data may indicate that the preferred methylated DNA target sites are unavailable due to being present in condensed heterochromatin regions [151,153]. While this may be a possibility for some gene loci depending on the cellular context, there is a notable loss of occupation at known and characterized ZBTB33 gene targets observed across several cell lines. Further, ZBTB33 also functions to directly up-regulate genes [129,130], which would be occurring within open euchromatin regions. An alternative explanation for the ChIP-seq data deviating from the other ChIP-based in cell studies is that this may be a consequence of technique-specific issues during ChIP-seq sample preparation. Indeed, several challenges in sample and library preparation for this widely used method, particularly for capturing occupation events for low abundance transcription factors, have been identified [154,155,156]. More recently, advanced methods for preparing ChIP-seq samples have become available (for example [157,158,159,160]) that may be able to clarify the global in cell genomic occupancies for this ZF MBP.

### 3.2. Zfp57

Genomic imprinting is a process by which gene expression from only the maternal or paternal allele is dictated, proper management of which is essential for normal development. In most instances, this monoallelic expression is directed by regulating DNA methylation patterns at CpG clusters termed imprinting control regions (ICRs) [161]. As such, it would seem reasonable that proteins that have evolved to have mCpG binding selectivity would function to mediate proper maintenance of this essential cellular process. Zfp57, a member of the KRAB domain family of ZF proteins, has increased expression levels during early embryonic development that tapers off upon embryonic stem cell differentiation [162]. During embryogenesis, ZFP57 has been shown to be required for DNA methylation maintenance at ICRs during both maternal and paternal DNA imprinting [163,164]. Indeed, the loss of Zfp57 in embryonic stem (ES) cells results in hypo-methylation of a number of imprinted gene regions, which cannot be re-established by re-introducing Zfp57 into this null background [164,165]. These findings are consistent with a series of ChIP-based experiments that showed Zfp57 preferentially binds to a CpG-containing hexanucleotide DNA sequence localized within ICRs in a methyl-dependent manner to facilitate proper epigenetic maintenance of chromatin during early embryonic development [164]. The fact that in vitro Zfp57 has been shown to have asymmetric DNA binding [99] suggests that it may be capable of binding hemi-methylated DNA within the cell to mechanistically maintain proper DNA methylation patterns at ICRs. Consistent with this idea, the localization of Zfp57 to its target methylated sites within ICRs induces an association with TRIM28 (also known as KAP1), which leads to further recruitment of DNMTs and UHRF1; all of which serves to maintain methylation during the imprinting process [161,164]. More recently, it has been demonstrated that cancer cells can take advantage of the ES cell function of Zfp57 to promote anchorage-independent growth, suggesting that this protein may have oncogenic potential when inappropriately overexpressed [166].

### 3.3. Klf4

Klf4 is a member of the specificity protein/Krüppel-like factor (SP/Klf) family of TFs [167] that is expressed in several tissues and functions in a number of core cellular processes. Importantly, Klf4 has also been shown to be one of four essential factors required for the induction of pluripotent stem cells [168]. Several reviews have summarized the tissue specific roles of Klf4 in development, normal cell function, and disease [169,170,171,172,173]. Klf4 has been shown in vitro to have comparable binding activity for recognizing DNA sequences harboring CpG, mCpG, or TpG steps [100,101]. It may be that this flexibility for motif recognition expands the mechanisms by which Klf4 is capable of functioning as a mediator of key developmental process [108]. Further, from ChIP-seq analyses in conjunction with bisulphite sequencing, it has been demonstrated that the moderate sensitivity of Klf4 for mCpG relative to CpG sites affords it the ability to localize at target loci regardless of methylation status [81,174]. However, it may be that preference for methylated loci is correlated with the cell context specific functions of this protein. Indeed, in mouse embryonic stem cells, Klf4 was found to localize to a moderate proportion of methylated DNA sites relative to non-methylated [81], while in glioblastoma cells, the occupancy at highly methylated sites was ~60% [174]. In contrast to the classical model of MBP TF binding at methylated promoters leading to repression, direct Klf4 occupation at a subset of its methylated target gene loci has recently been shown to recruit chromatin remodeling complexes that signal for the opening of chromatin and subsequent up-regulation of gene expression [174].

### 3.4. WT1 and Egr1

WT1 and Egr1 represent two ZF proteins that have highly divergent cellular functions. Egr1 cellular activities are induced within the cell in response to stress and various external stimuli [175]. In this capacity, Egr1 functions to regulate essential processes in both the nervous and cardiovascular systems [176,177,178,179]. WT1 is a transcription factor that plays a significant role in ensuring proper embryonic development of the genitourinary system [180,181,182]. WT1 can exist in a multitude of isoforms, and truncations or mutations of this essential protein have been correlated with several disease conditions [183,184]. Nevertheless, these two TFs have sequence conservation within three of their ZFs (ZFs 2–4 in WT1 and ZFs 1–3 in Egr1) that affords them the capability of binding to the same DNA consensus motif, 5′-GCG(T/G)GGGCG-3′. With two CpG sites, there is a significant potential for various forms of cytosine modifications to be present in this sequence within the cell at any given time.

This shared sequence is highly represented within the human genome [79], particularly at CGIs where Egr1 has been shown to in cell occupy utilizing ChIP-based studies [185,186]. It has generally been asserted that Egr1 functional target sites reside in promoter CGIs, which are typically non-methylated [153,186]. Thus, the relative indifference of Egr1 to occupy CpG relative to mCpG versions of its sequence motif would potentially pose an issue for ensuring that this protein is properly directed to its regulatory genomic targets. As such, mechanisms within the cell would be required to ensure that within the relatively short lifetime of the protein, required Egr1 transcriptional activities are not misappropriated by recruitment to non-functional methylated targets [79]. This is particularly important as it has been demonstrated that the combined number of functional and non-functional sites exceeds the number of typically expressed Egr1 protein molecules, and that a significant presence of ‘decoy’ sites regardless of methylation status kinetically impedes the ability of this protein to localize at relevant functional genomic elements [78,187]. It has recently been demonstrated in vitro that when present at sufficiently high concentrations, competitive occupation by a member of the MBD family of MBPs at methylated versions of the Egr1 DNA consensus serves to simultaneously protect Egr1 from binding at potential ‘decoy’ sites and kinetically drives Egr1 toward non-methylated target CGI regions [78]. WT1 has also been confirmed to localize to the shared Egr1 consensus motif in cell by ChIP-seq [188,189], however, the dependency of these occupation events on methylation status remain to be clarified.

It has been further demonstrated that WT1 and Egr1 have differential sensitivity to the oxidative derivatives of mC, including hydroxymethylC (hmC), formylC (fC),and carboxylC (caC) [102], which are generated in the cell through a series of oxidation reactions catalyzed by the ten-eleven translocation (TET) enzymes [190,191,192,193]. Mechanistically, this sensitivity difference appears to correlate with the presence or substitution of the core glutamate residue implicated in ZF MBP mC recognition. Specifically, both Egr1 ZF1 and ZF3 possess the key glutamate and arginine residues necessary for providing methyl sensitivity at the 5′- and 3′-GCG, respectively [102]. While ZF4 of WT1 also has the prerequisite arginine and glutamate residues, the glutamate position in ZF2 is substituted with a glutamine. This seemingly conservative alteration affords WT1 the ability to retain some binding capability for sequences harboring hmC, fC, and, in particular, caC, whereas the presence of these oxidative derivatives comparatively abolished binding for Egr1 [102]. Substitution of caC at only the 3′-GCG site resulted in an increased specificity for caC over mC by ZF2 of WT1, seemingly due to the ability of the glutamine residue to form a classical hydrogen bond with the caC carboxylate oxygen [102]. Intriguingly, it has been shown that in certain contexts, WT1 and Egr1 can function antagonistically [194]. It is interesting to hypothesize that this antagonistic behavior under certain cellular conditions may be in part correlated with the different sensitivities of these two proteins to the various forms of cytosine modifications.

### 3.5. CTCF and Interplay with Other ZF MBPs

CTCF is a highly expressed, well characterized ZF protein that binds to its specific core sequence through a tandem array of 11 Cys_2_His_2_ ZFs to elicit a number of cellular functions. Of central importance, CTCF is a key regulator of chromatin architecture that subsequently controls gene expression patterns. Recent high-resolution ChIP-exo experiments have refined the CTCF core motif to a 15-base pair sequence [195,196,197] that has the potential to acquire methylation on cytosines localized at positions 2 and 12 [198]. In cell, CTCF preferentially localizes to hypo-methylated regions, which serves to protect neighboring regions from acquiring DNA methylation. As an example, CTCF has been shown to protect the retinoblastoma (*Rb*) gene from epigenetic silencing [199]. Intriguingly, hyper-methylation of this promoter leads to a loss of CTCF binding and alternatively recruits ZBTB33, which induces methyl-dependent silencing of *Rb* [199]. While there is evidence to suggest that CTCF and ZBTB33 may compete for binding sites depending on the methylation status, these two TFs have also been shown to directly interact at the 5′ β-globin insulator region, which contains a KBS region proximal to the CTCF core binding site [200].

Recently, structural investigations have afforded insight for the impact of cytosine methylation at positions 2 and 12 in the CTCF core sequence [104]. As discussed above, CTCF ZF3 and ZF4 can contribute the key arginine and glutamate residues necessary to accommodate mCpG binding, and bind proximal to the cytosine at position 12. However, similar to WT1, Klf4, and Egr1, methylation of this site only marginally improved CTCF recognition of its core sequence. In contrast, methylation at the position 2 cytosine significantly impacted the ability of CTCF to bind to its core sequence. Inspection of the residues in ZF7, which is centered at cytosine 2, shows that the key arginine is present, but the glutamate residue is replaced with an aspartate. There is increasing evidence to suggest that while glutamate can provide sensitivity to mC, an aspartate substitution within ZFs confers selectivity for unmodified cytosine [100,107,108,201,202]. This is likely due to the inability of the shorter aspartate side chain to adopt an alternative conformation, which leads to a steric interference with the mC methyl group. Also analogous to Zfp57, Klf4, WT1, and Egr1, CTCF binding at the second cytosine position is asymmetric. This has interesting implications in that the genomic region of the H19 locus that encompasses the second cytosine position overlaps with the Zfp57 consensus site. The methyl sensitivity of CTCF and Zfp57 occurs on opposite strands within this region, suggesting that the status of cytosine modification could mediate binding interactions for these two TFs at this genomic site [104,108]. The cellular consequence of this disparate sensitivity to methylation at the two cytosine positions remains to be fully evaluated, but could offer an additional layer of epigenetic control for directing CTCF activities.

## 4. Concluding Remarks

In recent years, several structures of ZF MBPs in complex with their methylated DNA targets have become available, affording mechanistic insight into the commonalities and differences in the mode of mCpG recognition between family members. Of particular note, all of the structures to date show the common usage of a mC-Arg-G triad in mCpG recognition as well as a key glutamate that makes specific contacts with the mC. However, it is becoming evident that the number and nature of these contacts between the glutamate and mC dictates the extent for which these proteins are capable of distinguishing between mCpG relative to CpG sites. Further, substitution of the glutamate residue appears to afford increased sensitivity of ZF TFs for either unmodified or various oxidized variants of mC. In addition, recent findings from the C-terminal ZFs of ZBTB38 have demonstrated that a lysine residue can act as a surrogate for the key arginine residue, suggesting that alternative modes of mCpG recognition by ZF proteins are available. Indeed, it is important to reiterate that a number of additional ZF proteins have been identified from recent high-throughput studies to potentially have preferential mCpG binding capacity. Characterization of these additional ZF proteins may consequently illuminate additional mechanisms for mCpG recognition by this common protein scaffold. Finally, advances for in cell high-throughput sequencing strategies have begun to afford a more detailed understanding for how mCpG sensitivity correlates with the intricate biological functions elicited by the ZF MBPs in both normal cell function as well as various disease contexts.
